# Thoracic endovascular aortic repair for recurrent stroke due to atheromatic plaque in the proximal descending aorta: a case report

**DOI:** 10.1186/s40792-021-01187-7

**Published:** 2021-04-28

**Authors:** Masafumi Hashimoto, Yoshikazu Nakano, Yusaku Tamura

**Affiliations:** 1 Division of Cardiovascular Surgery, Chibaken Saiseikai Narashino Hospital, Social Welfare Organization Saiseikai, Imperial Gift Foundation Inc., 1-1-1 Izumi chou Narashino city, Chiba, 275-8580 Japan; 2 Division of Neurology Chibaken Saiseikai Narashino Hospital, Social Welfare Organization Saiseikai, Imperial Gift Foundation Inc., Chiba, Japan

**Keywords:** Atheromatic plaque, Idiopathic thrombocytopenia, Retrograde aortic flow, Stroke, Thoracic endovascular aortic repair

## Abstract

**Background:**

Diastolic retrograde flow in the descending aorta (DAo) may occur in the presence of atherosclerosis and may be overlooked as a mechanism of retrograde embolization in patients with stroke. We performed thoracic endovascular aortic repair (TEVAR) in a patient with recurrent cerebral infarctions for treatment of aortic aneurysm with atheromatic plaque, which was considered as the source of embolism.

**Case presentation:**

A 56-year-old man with a history of idiopathic thrombocytopenia and hypertension was referred to our hospital with paralysis of the right upper and lower limbs. Multiple cerebral infarctions were found and treated; however, 1 month later, another cerebral infarction developed. A small saccular aortic aneurysm with plaque was found beyond the left subclavian artery, and this site was deemed as the source of embolism. We performed TEVAR to prevent further recurrence of cerebral infarctions. No cerebral infarctions were observed 6 months post-operation.

**Conclusions:**

TEVAR is a useful treatment for not only aortic aneurysm and dissection, but also cerebral infarctions caused by an embolic source proximal to the DAo due to retrograde aortic blood flow.

## Background

An increasing body of evidence suggests that diastolic retrograde flow in the descending aorta (DAo) may frequently occur in the presence of atherosclerosis and become an overlooked mechanism of retrograde embolization in patients with stroke [1, 2]. Retrograde flow from complex DAo plaques are frequently detected in both determined and cryptogenic stroke and could explain the cause of embolism in all brain territories [1]. We performed thoracic endovascular aortic repair (TEVAR) for recurrent cerebral infarctions due to a distal arch aortic aneurysm with atheromatic plaque, which was considered as the source of embolism.

## Case presentation

**Fig. 1:**
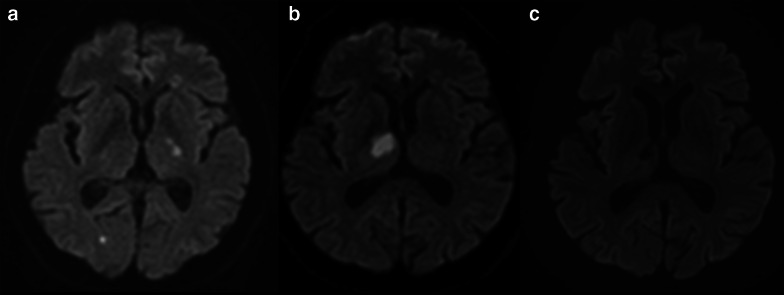
Brain diffusion-weighted magnetic resonance images. **a** multiple cerebral infarctions (right occipital lobe, left internal capsule putamen). **b** Cerebral infarction recurrence (internal capsule of the right thalamus) seen 1 month later. **c** No cerebral infarction 6 months postoperatively

A 56-year-old man with a history of idiopathic thrombocytopenia (ITP) and hypertension was referred to our hospital with paralysis of his right upper and lower limbs. The patient had no history of obvious arrhythmias, such as atrial fibrillation. Brain magnetic resonance imaging (MRI) showed multiple cerebral infarctions (Fig. 1a). One month later, while determining the source of the embolus, the patient developed another cerebral infarction (Fig. 1b). Blood examination revealed only 1.1 × 10^4^/μL platelets (2.7 × 10^4^/μL using citrate blood sampling) because of ITP; therefore, antiplatelet therapy could not be administered. There were no arrhythmias (including atrial fibrillation), and transthoracic echocardiography showed no obvious intracardiac thrombus. Transesophageal echocardiography also found no obvious embolic source structure, patent foramen ovale, or decrease in the blood flow of the left atrial appendage. The bubble test results were also negative.

**Fig. 2:**
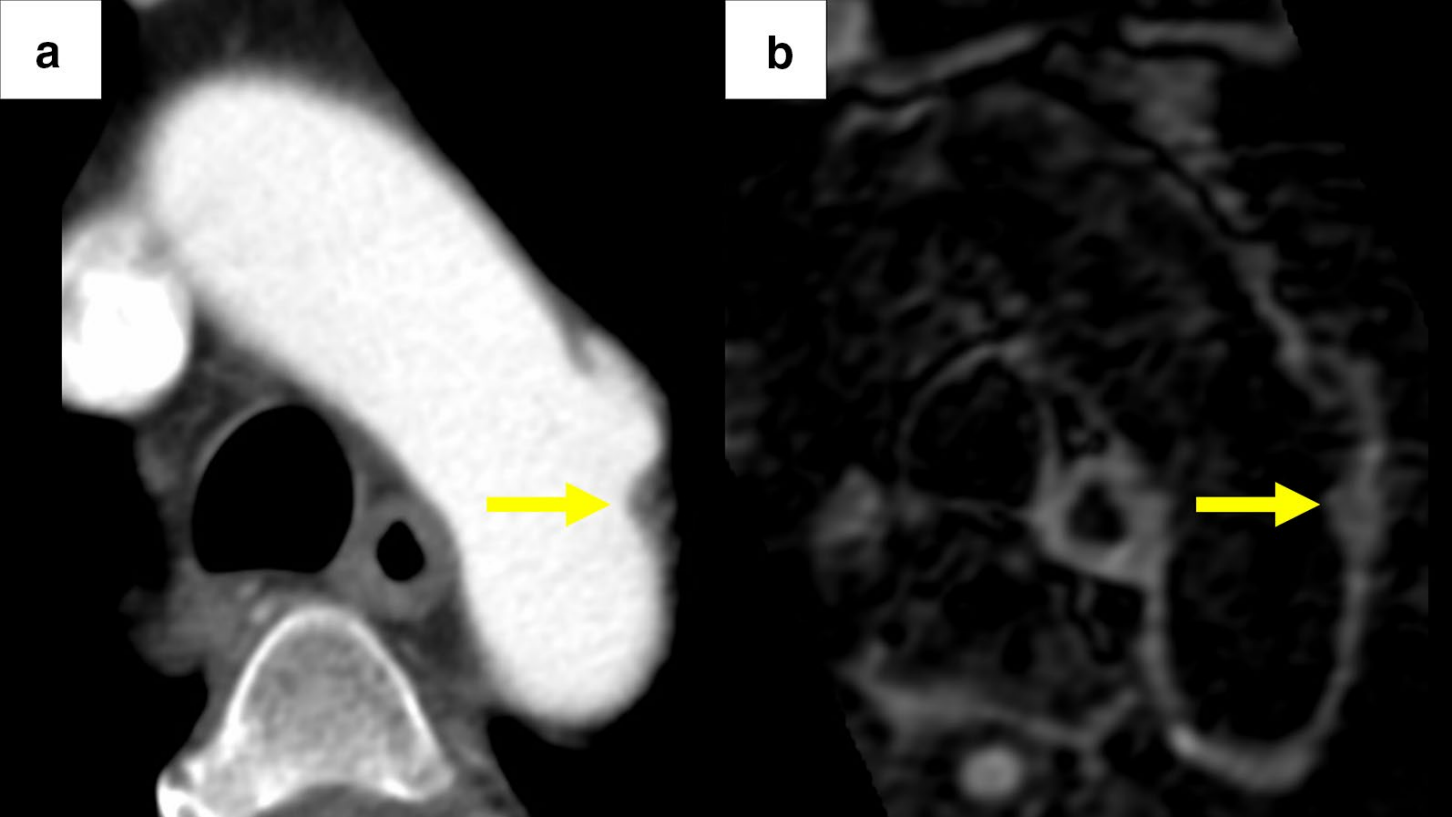
There is a small saccular aortic aneurysm on the distal arch with the atheromatic plaque. The saccular aneurysm was approximately 35 mm in diameter and the atheromatic plaque thickness was approximately 4 mm. **a** Computed tomography angiogram images and **b** magnetic resonance images

**Fig. 3:**
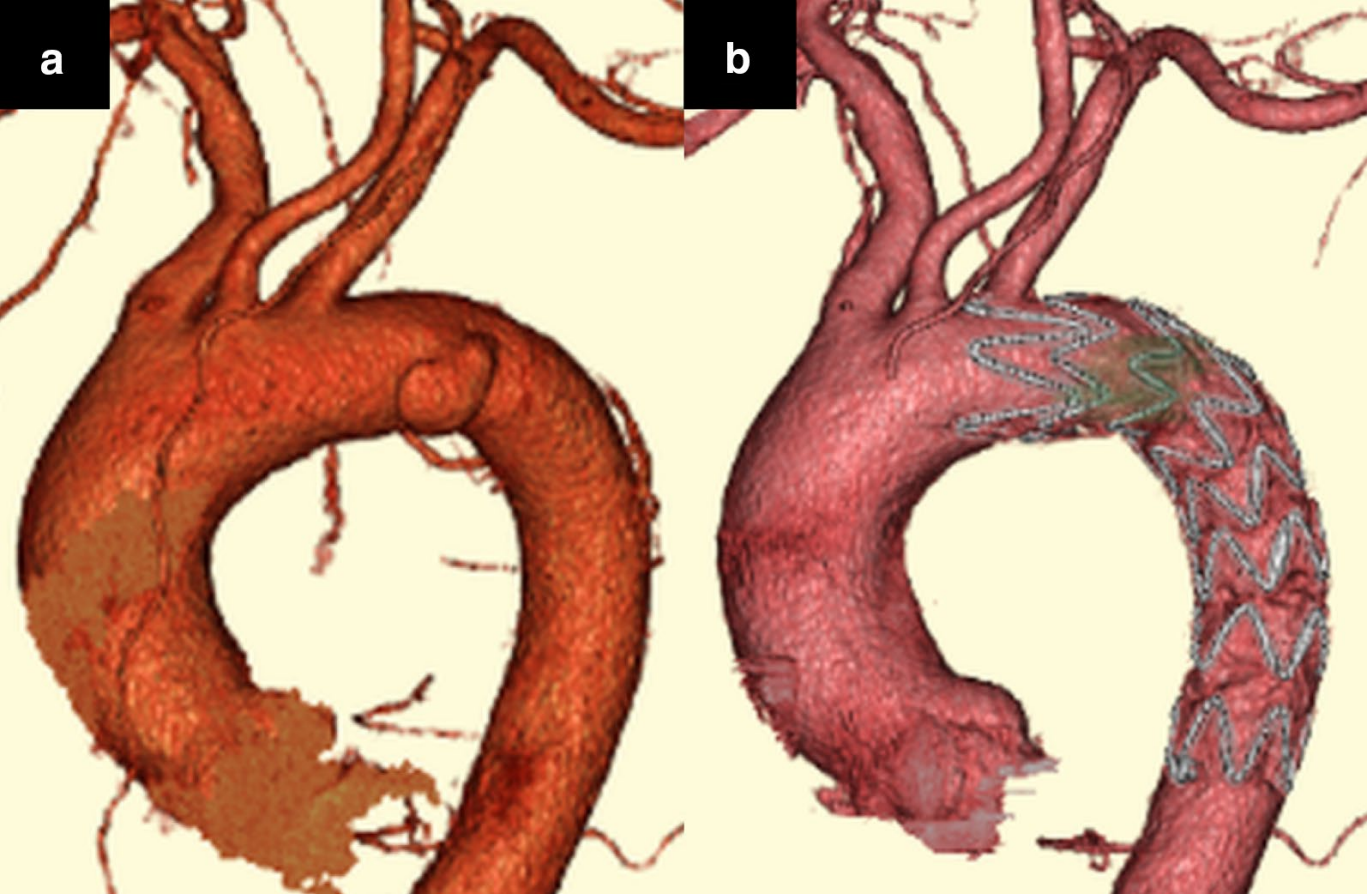
Computed tomography angiogram images. **a** Thoracic aortic aneurysm (35 mm) approximately 15 mm from the left subclavian artery with atheromatic plaque (preoperative). **b** Postoperative confirmation of absence of endoleaks

Computed tomography angiography (CTA) and MRI revealed a small saccular DAo aortic aneurysm with atheromatic plaque beyond the left subclavian artery (Fig. 2a, b, and Fig. 3a). No other visible embolic source was found. Thus, the DAo site was considered the source of embolism. TEVAR was planned to prevent stroke recurrence. Preoperatively, immunoglobulin was administered at 30 g/day for 4 days, and the platelet count improved to 22.6 × 10^4^/μL (24.0 × 10^4^/μL using citrate blood sampling).

TEVAR was performed in zone 3 using a Relay Plus (Terumo Corporation, Tokyo, Japan; AU-3414530). Endoleaks were not observed during intraoperative imaging; this was confirmed postoperatively using CTA (Fig. 3b).

After the operation, the motor function recovered, and the patient could walk independently, but the attention disorder and memory impairment persisted. Therefore, the patient was transferred to another hospital for rehabilitation. Six months after the operation, no cerebral infarctions were observed (Fig. 1c).

## Conclusions

Until recently, flow reversal in the thoracic aorta was believed to be a sign of aortic regurgitation. However, studies using transesophageal echocardiography or 4D-MRI showed aortic flow reversal without aortic regurgitation [1, 3, 4]. Evidence has indicated that arterial stiffness and wall thickness, which are associated with aging and atherosclerosis, can lead to flow reversal due to a mismatch between peripheral and central arterial stiffness [3, 4]. Typically, retrograde blood flow originates in the first 20–30 mm of the proximal DAo and can reach all brain-supplying arteries [2]. Thus, atheromas at this site may cause embolic stroke in any brain territory [2].

Since the infarct site was different between the first and recurrent strokes, there was a high possibility of embolic cerebral infarction. Full-body examination did not reveal the source of embolism other than the atheromatic plaque, which was located approximately 15 mm from the left subclavian artery on the proximal side. If 4D-MRI could be performed, it would be clearer that the cause of the stroke was the atheromatic plaque, but unfortunately it was not possible at our institution. Despite the small size of the aneurysm, TEVAR was performed because of the history of ITP and the patient’s ineligibility to undergo antiplatelet therapy.

TEVAR is a useful treatment for cerebral infarction caused by an embolic source proximal to the DAo due to retrograde aortic blood flow. We hope that this case report will help in the treatment of cryptogenic stroke.

## Abbreviations

Dao: Descending aorta
CTA: Computed tomography angiography
MRI: Magnetic resonance imaging
TEVAR: Thoracic endovascular aortic repair
ITP: Idiopathic thrombocytopenia
